# ChartLine: Automatic Detection and Tracing of Curves in Scientific Line Charts Using Spatial-Sequence Feature Pyramid Network

**DOI:** 10.3390/s24217015

**Published:** 2024-10-31

**Authors:** Wenjin Yang, Jie He, Qian Li

**Affiliations:** 1 School of Computer and Communication Engineering, University of Science and Technology Beijing, Beijing 100083, China; d202110385@xs.ustb.edu.cn; 2 Beijing Advanced Innovation Center for Materials Genome Engineering, University of Science and Technology Beijing, Beijing 100083, China; hejie@ustb.edu.cn; 3 Beijing Key Laboratory of Knowledge Engineering for Materials Science, Beijing 100083, China; 4 Shunde Graduate School, University of Science and Technology Beijing, Foshan 528300, China; 5 Institute for Advanced Materials and Technology, University of Science and Technology Beijing, Beijing 100083, China

**Keywords:** curve detection, self-attention, feature pyramid, BiLSTM

## Abstract

Line charts are prevalent in scientific documents and commercial data visualization, serving as essential tools for conveying data trends. Automatic detection and tracing of line paths in these charts is crucial for downstream tasks such as data extraction, chart quality assessment, plagiarism detection, and visual question answering. However, line graphs present unique challenges due to their complex backgrounds and diverse curve styles, including solid, dashed, and dotted lines. Existing curve detection algorithms struggle to address these challenges effectively. In this paper, we propose ChartLine, a novel network designed for detecting and tracing curves in line graphs. Our approach integrates a Spatial-Sequence Attention Feature Pyramid Network (SSA-FPN) in both the encoder and decoder to capture rich hierarchical representations of curve structures and boundary features. The model incorporates a Spatial-Sequence Fusion (SSF) module and a Channel Multi-Head Attention (CMA) module to enhance intra-class consistency and inter-class distinction. We evaluate ChartLine on four line chart datasets and compare its performance against state-of-the-art curve detection, edge detection, and semantic segmentation methods. Extensive experiments demonstrate that our method significantly outperforms existing algorithms, achieving an F-measure of 94% on a synthetic dataset.

## 1. Introduction

**Figure 1:**
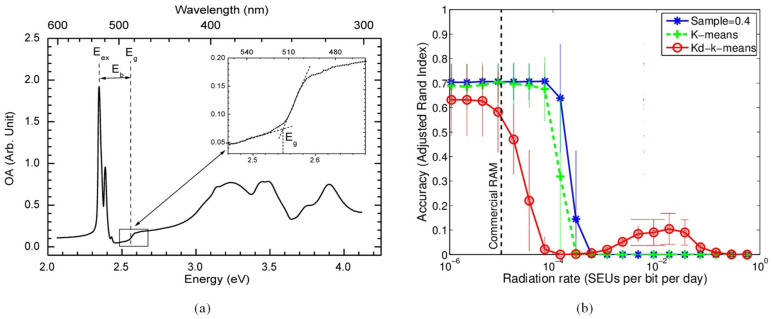
Two samples with variable curve types and indistinguishable noise and curves. Both images are from the FigureSeer dataset, (**a**) is a gray image and (**b**) is color image.

Line chart images, such as in Figure 1, are widely found in scientific papers. These charts are created for data visualization and analysis, but the raw data in the charts may not be directly accessible. Automatically extracting those data can bring tremendous benefits in scientific document processing, redesigning charts, and enhancing the reading experience for the visually impaired [1,2,3,4]. Especially in the field of new materials research, mining the data in a paper can accelerate the development of new materials [5]. The line chart in a paper contains a large amount of raw data. It is essential to analyze line charts. Mining a chart’s data requires understanding the spatial and semantic relationships of the chart components [3,6]. However, those relationships are not only dependent on the type of chart but also on the chart component type and position. Factors such as chart layout, curve overlap, occlusion, and compression artifacts are significant challenges for data reconstruction [7,8]. Due to the variety of image styles and curve types, curve detection becomes more difficult. Existing curve extraction systems, such as ReVision [9], require a clean image background with no noise interference. Although it is possible to parse a wide range of chart types and obtain raw data, more is needed for the line chart. In fact, until now, the automatic extraction of curves from line chart images with complex backgrounds has been an enormous challenge.

Extracting the raw data from the line chart can be divided into two steps: the first is to extract the curve from the image, and the second is to extract the points from the curves. A line chart image is visually a linear or curve structure, and curve detection is currently a fundamental problem in computer vision. The curve can be understood from two perspectives. From a local perspective, it is a linear object with a certain width. From a global perspective, it looks like the edge of an object in an image, and usually has a large difference compared to the image background. Therefore, there are two main types of curve detection methods: image segmentation-based and edge detection-based methods. Ideally, if the curve is continuous and separable from the background, then conventional edge detection and image segmentation algorithms are theoretically able to detect the curve effectively.

However, in practical application, the grid lines may disturb the curve, especially when the image is not a color image or the background is similar to the curve, making the natural curve challenging to distinguish. In addition, when the curve type in the image is dotted or a dash–dot, the curve extraction becomes more difficult. In addition to the factors of the curve itself, some auxiliary information on the image, such as arrows and text, will also interfere with the correct detection of the curve, as shown in Figure 1. These complications often degrade the performance of or even fail traditional curve detection methods.

Recently, deep convolutional neural networks (DCNNs) have been widely used in the field of computer vision (e.g., image classification [10], object detection [11,12,13,14], image segmentation [15,16], etc.). Curve detection methods based on deep learning are also widely used for edge detection [17,18], boundary segmentation [19], defection detection, etc. These deep convolution architectures acquire multi-scale features of the input image layer by layer, enabling the separation of the linear structure and the background. CS^2^-Net [20] was proposed to implement medical image curve structure segmentation, which can adaptively fuse the global features of space and channels to improve cell segmentation performance effectively. Numerous other structures based on U-Net show that the decoder layers in the network can effectively enhance semantic segmentation. In addition, the use of recurrent neural networks on tasks with long-term dependencies can also improve the model’s performance, e.g., [3] combined convolution and recurrent neural networks to improve the data extraction of curves effectively. However, these methods must filter the noise better, and the detected curves are coarser or even discontinuous.

Inspired by these observations, we design a curve detection network based on the spatial-sequence attention module, which can capture the rich contextual dependencies. We build an encoder–decoder structure based on ResNet. Unlike CS^2^-Net, we introduce a Spatial-Sequence Attention Feature Pyramid Network (SSA-FPN) between the encoder and decoder. It contains two sub-modules—Spatial-Sequence Fusion (SSF) and Channel Multi-Head Attention (CMA). SSF can capture the boundary features of curves and fuse local and global information and can enhance the continuous dependence of the curve. CMA can adaptively fuse curves and background information to improve curves’ differentiation ability effectively. In summary, our contributions are as follows:

(1) We design a novel curve structure detection network (ChartLine) based on Long Short-Term Memory (LSTM) and self-attention modules, which can enhance the discriminative power of curve feature representations for curve segmentation.
(2) A Spatial-Sequence Attention Feature Pyramid is introduced to ChartLine, which can enhance the network to capture long-range dependence and fully use the multi-channel space for feature representation. It can effectively separate the curve from the background and enhance the continuity of the curve.
(3) A new loss function is proposed to solve the problem of having fewer curve samples in the chart image during training to improve the precision and recall of the model.
(4) Four datasets are collected for model training and performance evaluation, two of which are used for training the network and testing, while the other two are used for testing only. These datasets are shared with the community to facilitate research into line chart parsing and data restoration. A large number of experiments are conducted and prove the effectiveness of the proposed method.

## 2. Related Work

### 2.1. Cure/Edge Detection

Cure/edge detection [21,22,23] is a fundamental problem in computer vision, widely used in tasks such as object recognition and image segmentation. Classical edge detection algorithms, such as the Canny edge detector [21], identify edges by calculating pixel intensity gradients and detecting regions with significant intensity changes. With the development of convolutional neural networks (CNNs), models like HED [17,24], FCN [16], and RCF [18] have further improved the accuracy and robustness of edge detection. HED leverages multi-scale feature fusion, achieving excellent performance in edge detection tasks, while RCF enhances edge detection by using side outputs from each convolutional layer, capturing richer edge features. In addition, deep learning-based curve detection has achieved promising results in other fields, such as video segmentation [25] and medical image segmentation [26]. CS^2^-Net [20] was proposed to implement medical image curve structure segmentation, which can adaptively fuse the global features of space and channels to improve cell segmentation performance effectively. While these methods excel in traditional edge detection, they are not entirely suitable for detecting curves in line charts. Edge detection primarily focuses on identifying object boundaries or contours in an image, whereas line chart curve detection requires the precise extraction of curves, particularly with regard to their continuity and accuracy. Edge detection methods are typically designed to capture high-contrast edges, but the curves in line charts are often smooth and lack distinct boundary features, making gradient-based edge detection techniques less effective in capturing all the details of the curves.

### 2.2. Chart Line Detection

The curves in line chart images can be thin, overlapping, and have diverse drawing styles, resulting in challenging curve extraction. Earlier work [27] used binary images and refinement to extract single lines in images. Ref. [28] used linear and nonlinear regression algorithms to extract data points in images and fit them to a curve. Lu [29] proposed a method based on the original chain encoding and curve reconstruction algorithm for multiple actual lines. FigureSeer [30] provides a comprehensive analysis of graphs and converts curve extraction to an optimal path-finding problem, using dynamic programming to achieve curve extraction. Specifically, first, the legend is parsed to determine the number of curves in the image in total, and subsequently, each curve is tracked individually according to the legend. ChartOCR [31] proposes a hybrid architecture based on deep learning to extract the key points in the image by modifying the CornerNet and using a convolution embedding layer to make the points belonging to the same curve as close as possible and the points of different curves as far away as possible. Finally, the detection of curves is achieved by a union–find algorithm. LineEX [32] extends ChartOCR by using a transformer in feature extraction, further improving the accuracy of key point extraction for curve charts. While these key-point-based extraction methods work well for some simple images, their accuracy drops dramatically for more complex situations such as partially occluded curves or point-linear curves.

## 3. Method

### 3.1. Network Architecture

**Figure 2:**
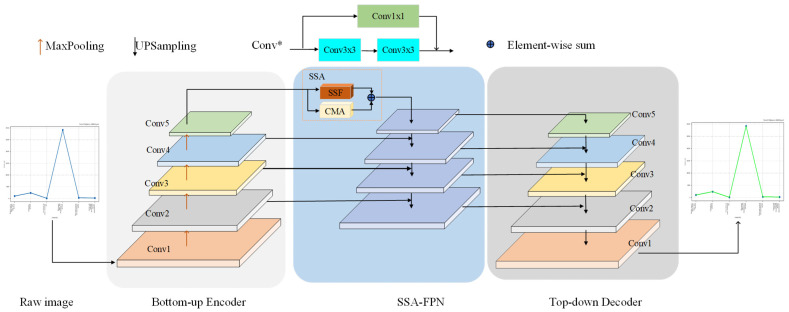
An illustration of the ChartLine network. The encoder and decoder are used to construct multi-scale features, the attention feature pyramid is used to capture the long dependencies of the curves and adaptively distinguish between the foreground and background, and the decoder’s output is a curve probability map. Conv* represents Conv1–5.

ChartLine is inspired by CS^2^-Net [20]. ChartLine is a deep hybrid network architecture designed for pixel-wise curve detection. As shown in Figure 2, it contains an encoder network, a corresponding decoder network, and an SSA-FPN module. The encoder consists of five encoder layers and five down-sample layers. Each encoder layer consists of two convolution layers and a skip connection. The decoder and encoder layers have a similar structure. Each decoder layer has a corresponding layer in the encoder layer. The difference is that the first encoding layer produces a multi-channel feature map. In contrast, the corresponding decoding layer produces a one-dimensional channel feature map because we only focus on the curve, not the background. Therefore, we only model the curve. The SSA-FPN module can adaptively aggregate contextual information over a long range to enhance the detailed representation of features.

Given a line chart image or feature map, a batch normalization and nonlinear activation function are applied to the feature map after the convolution operation. A max-pooling layer with a step size of 2 is used in each encoding layer to scale down the feature map, with translation invariance in local space. A skip connection is used in the encoder structure to prevent gradient disappearance, and after multi-scale feature extraction, the input image is finally scaled to 1/32 of the original size. The encoded features are fed into the SSA-FPN. The encoded features of the fifth layer are passed into the two parallel submodules of the SSA-FPN to enhance the intra-class responsiveness and inter-class differentiation of the model. We apply a 3 × 1 convolution layer to obtain the spatial features in the horizontal direction and use a bidirectional LSTM to convert the spatial features into sequence features. A similar process is used for the vertical direction to obtain the sequence features in the vertical direction. The features in both directions are combined into one sequence feature using a concat layer.

Finally, a channel MLP is used to fuse the feature information and reduce the dimension of the features. A multi-head attentive mechanism is constructed to distinguish the curve from the background better to achieve adaptive separation of the curve from the background. Specifically, we first perform dimensional transformation and generate multiple channel attention matrices. Next, we perform matrix multiplication between the attention matrix and the deformed features. Finally, we perform an element summation operation on the resultant matrix of the above multiplication and the original features and stitch the resulting features together with the spatial features. To enhance the detail and hierarchical features of the curves, top-down fusion encodes the features, inspired by [33,34]. As shown in Figure 2, the features of the hybrid module are merged with those of the fourth layer of the coding layer and subsequently used as input for the next layer, repeating the process from top to bottom until the bottom layer. The fusion of multi-scale coding features facilitates the acquisition of more coarse-grained and fine-grained curve features. Finally, the multi-scale feature map generated by the feature pyramid is parsed top-down using a decoder to output the final curve probability map. Since the deep convolutional layer has a larger perceptual field than the shallow convolutional layer, it can obtain more contextual information. The top-down fusion of multi-scale features helps improve curve detection accuracy.

### 3.2. Spatial-Sequence Fusion (SSF) Module

**Figure 3:**
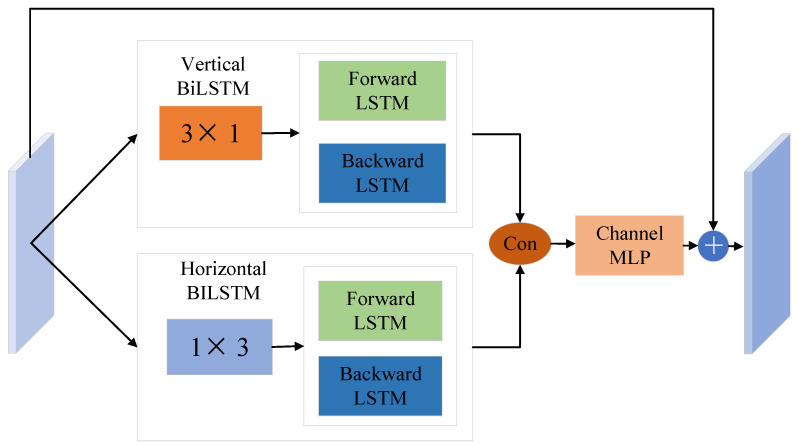
The details of the Spatial-Sequence Fusion module.

The global feature is necessary for curve detection, which can be obtained by capturing contextual information from the remote. Many works [16,35,36] have shown that local features generated by FCN may lead to the misclassification of targets and boundaries. To better model the prosperous contextual relationships, we transformed the spatial features of the curves into sequence features. We enhanced the curve representation capability of the model by using the spatial-sequence module to model the curve-length dependencies. Moreover, we capture the boundary features of the curve using 1 × 3 and 3 × 1 convolutions. Figure 3 shows the overall structure of the Spatial-Sequence Fusion module; it contains a horizontal spatial-sequence module and a vertical spatial-sequence module. Each module contains a convolution and a bidirectional LSTM. A channel MLP is then used to fuse the sequence features in both directions and restore the dimensionality of the features to their original dimensions.

The LSTM is a special type of recurrent neural network (RNN) with the capability of learning long-range dependence. The LSTM contains input gates, forget gates, and output gates. The input gate *i_t_* controls the input information, the forget gate *f_t_* prevents the former unit state *c_t_*_−1_ from being forgotten, and the output gate *o_t_* controls the cell output *h_t_* of the current cell state *c_t_*. The LSTM is formulated as follows:

(1) $$f_{t}=\sigma_{g}\left(W_{f}x_{t}+U_{f}c_{t-1}+b_{f}\right);$$

(2) $$i_{t}=\sigma_{g}\left(W_{i}x_{t}+U_{i}c_{t-1}+b_{i}\right);$$

(3) $$o_{t}=\sigma_{g}\left(W_{o}x_{t}+U_{o}c_{t-1}+b_{o}\right);$$

(4) $$c_{t}=f_{t}⊙c_{t-1}+i_{t}⊙\sigma_{c}\left(W_{c}x_{t}+b_{c}\right);$$

(5) $$h_{t}=o_{t}⊙\sigma_{c}\left(c_{t}\right),$$

where $\sigma_{g\;}$ is a sigmoid function, $\sigma_{c}$ is a hyperbolic function (tanh), and ⊙ denotes the dot product. The BiLSTM consists of a forward LSTM and an inverse LSTM, which is profitable for sequences where mutual dependencies are expected. The output of the BiLSTM is obtained as follows:

(6) $$\vec{h_{f}}={LSTM}_{f}(\vec{x});$$

(7) $$\overset{\leftarrow }{h_{b}}={LSTM}_{f}(\overset{\leftarrow }{x});$$

(8) $$h=concat(\vec{h_{f}},\overset{\leftarrow }{h_{b}}),$$

where $\vec{x}$ is the input series, $\overset{\leftarrow }{x}$ is the inverse sequence of $\vec{x}$, $\vec{h_{f}}$ is the output of the forward LSTM, and $\overset{\leftarrow }{h_{b}}$ is the inverse sequence of $\vec{h_{f}}$.

For better access to spatial information and global dependence, we design a spatial-sequence module based on LSTM. Let $F\in R^{B\times C\times H\times W}$ denote a feature map, where B, H, W, and C represent the batch size, height, width, and the number of channels, respectively. First, the horizontal direction of the feature map is processed as follows:

(9) $$F_{:,w,:}^{v}=BiLSTM({Conv}_{v}(F)),$$

where *Conv_v_* contains a 1 × 3 convolution. Normalization (BN) and a nonlinear activation function (ReLU) are used to encode the spatial features in the vertical direction, as shown at the top of Figure 2, and $F_{:,w,:}^{v}\in R^{H\times 2D}$ denotes the sequence feature in the vertical direction; here N is equal to B*W. D is the number of BiLSTM hidden layers. A similar approach is used for the horizontal direction feature encoding, as follows:

(10) $$F_{h,:,:}^{h}=BiLSTM({Conv}_{h}(F)),$$

where *Conv_h_* contains a 3 × 1 convolution. Normalization (BN) and a nonlinear activation function (ReLU) are used to encode the spatial features in the horizontal direction, as shown at the bottom of Figure 3, and $F_{:,h,:}^{h}\in R^{W\times 2D}$ denotes the sequence feature in the horizontal direction. Finally, we convert $F_{:,w,:}^{v}\in R^{H\times 2D}$ into $F^{v}\in R^{H\times W\times 2D}$ and convert $F_{:,h,:}^{h}\in R^{N\times W\times 2D}$ into $F^{h}\in R^{H\times W\times 2D}$. Finally, the channel information is fused by a multi-layer perceptron. These processes are formulated as follows:

(11) $$F^{\prime }=concat(F^{v},F^{h}),$$

(12) $$F^{\prime \prime }=ChannelMlp\left(F^{\prime }\right),$$

where $F^{\prime \prime }\in R^{C\times H\times W}$ and ChannelMlp is stacked by two layers of the perceptron.

### 3.3. Channel Multi-Head Attention (CMA) Module

In general, the high-level channel feature can be regarded as the convolution response to specific classes, and the responses of different semantics are correlated. Many works [35,36] have proposed a channel attention module to exploit the inter-dependence of different channel feature maps. Unlike those works, we do not utilize convolution layers to embed the features before calculating the correlation of different channel maps; only by using reshaping to rearrange the feature maps can the relationships of different channel maps be preserved. This way, we could emphasize inter-dependent feature maps and improve the feature representation of different channel semantics.

**Figure 4:**
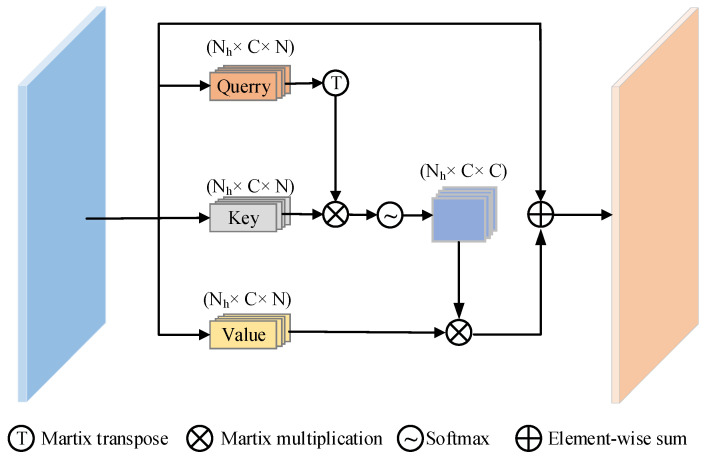
The details of the Channel Multi-Head Attention module.

Self-attention was first proposed in [36] to solve the long-range semantic dependence problem of sequence models for natural language processing. It has now been extended to computer vision applications with excellent results in object detection, image recognition, etc. However, unlike those works that divide images into multi-tokens, we deform the final encoder layer output as a token, which can reduce a lot of computation time and resource requirements. The network structure of Channel Multi-Head Attention module is shown in detail in Figure 4. To calculate the attention matrix $A\in R^{C\times C}$ of the original feature $F\in R^{C\times H\times W}$, we first reshape *F* to $R^{C\times N\times M}$ (*M* is the number of self-attention heads). After that, we multiply *F* and the transpose matrix of *F* (named *F^T^*); in this way, similar semantic features will promote each other, while different semantic features will inhibit each other. As a final step, we apply a SoftMax layer to obtain M channel-wise attention matrices $A_{m}\in R^{C\times N}$:

(13) $$A_{m}\left(i,j\right)=\frac{\exp\left(F_{m}\cdot F_{m}^{T}\right)}{\sum_{i,j=1}^{C}\exp\left({F_{m}}_{i}\cdot {F_{m}}_{j}^{T}\right)},$$

(14) $$\begin{array}{c} A\left(i,j\right)=Concat\left(A_{0},A_{1},\cdots ,A_{M}\right), \end{array}$$

where $A_{m}\left(i,j\right)\in R^{C\times N\times M}$, and we reshape $A_{m}$ to $A\in R^{C\times H\times W}$, which measures the degree of influence of the *i*th channel on the *j*th channel. After obtaining the channel-wise matrix, we multiply A with the transpose of *F* and deform the result to $R^{C\times H\times W}$.

### 3.4. Hybrid Loss (HL) Function

Unlike other semantic segmentation tasks, there are only two types of curve detection. Curve segmentation can be regarded as a binary classification problem. Binary cross-entropy loss is mainly used to assess prediction errors in many binary classification tasks. Generally, curve pixels are a minority in line chart images, which results in model training with unbalanced positive and negative samples. Some works [18,27] in the field of linear structure detection have proposed adding larger weights to a few classes to solve the sample imbalance problem. However, in curve detection, we experimentally find that adding larger weights to the curve samples will yield more false positives. In order to better solve the above problem, we propose a hybrid loss function to evaluate the prediction results.

We propose an improved Dice loss function (named ImDice) based on the ensemble similarity measure function, which takes values from 0 to 1, with higher values indicating higher similarity. Dice loss was first proposed in VNet [19] and then widely used in medical image segmentation, such as in [20], etc. However, these works only consider the case of positive samples, which will lead to more false positive cases; so, we make parameter adjustments for negative samples, which is an effective way to improve accuracy and address sample imbalance to some extent. Therefore, we define the improved Dice loss as:

(15) $$L_{ImDice}=1-\frac{2\sum_{i}^{I}p*g_{t}+\varepsilon }{\sum_{i}^{I}p^{2}+\sum_{i}^{I}g_{t}^{2}+\varepsilon }-\frac{2\sum_{i}^{I}\left(1-p\right)*\left(1-g_{t}\right)+\varepsilon }{\sum_{i}^{I}{\left(1-p\right)}^{2}+\sum_{i}^{I}{\left(1-g_{t}\right)}^{2}+\varepsilon },\;$$

where p is the predicted result obtained from the sigmoid function. The sigmoid function can transform the feature map into a curve probability map. $g_{t}$ is the ground truth label. The variable “I” represents the number of pixels in the image, while ε refers to the Laplace smoothing coefficient, and ε is set to 1. The introduction of ε has two main advantages: (a) it ensures that the denominator is not zero when both the number of predicted curve pixels and the number of true curve pixels are zero, and (b) it helps to reduce the risk of overfitting.

However, using only ImDice loss as a loss function causes the training process to be unstable to the extent that it is difficult to converge; so, to speed up the model convergence, we use improved cross-entropy loss (named ImBCE) in combination with ImDice. We found that the direct use of cross-entropy loss will reduce the recall of the model, which is due to the fact that the curve pixels are ten thousandth of the overall pixels, leading to a more negative sample bias in the model training. Therefore, we improved the cross-entropy loss, which can effectively improve the precision and recall of the model by correcting the samples that are incorrectly predicted during the training process, as follows:

(16) $$\begin{array}{c} L_{ImBce}=\sum_{i}^{I}{\alpha p}_{t}^{\gamma }\log\left(p\right)+p_{t}^{\gamma }\log\left(1-p\right), \end{array}\;$$

(17) $$p_{t}=\left\{\begin{array}{c} \left(1-p\right)*g_{t},\;{\;\;\;\;\;\;\;\;\;\;\;\;\;\;\;\;\;\;if\;g}_{t}=1,\\ {p*(1-g}_{t}),\;\;\;\;\;\;\;\;\;\;\;\;\;\;\;otherwise,\; \end{array}\right.,\;$$

where α and γ are both constants. Then, the total loss can be formulated as:

(18) $$L={\beta L}_{ImBce}+L_{ImDice},$$

where β is a constant that is greater than 1. This is because the cross-entropy loss tends to zero easily; hence, adding a coefficient helps the model to converge better.

## 4. Experiments and Results

### 4.1. Implementation Details

We use the deep learning architecture PyTorch, which is very well-known in the community, to implement our network. We use residual connections throughout the network to solve the gradient disappearance problem during training and batch normalization to accelerate the convergence of the model. We use bilinear interpolation for up-sampling in the feature pyramid process and deconvolution in the decoding network. The entire network is initialized with random values obeying a (0,1) normal distribution for the weights, and the bias is initialized to 0. During training, the global initial learning rate is 1 × 10^−4^, and the learning rate is dynamically adjusted by a sine function. We use Adam to update the weight parameters with a batch size of 32. The model is saved every two epochs, and one epoch means that all data are trained once. All experiments in this paper are implemented using four NVIDIA GeForce 3090 machines, and the models are trained in a distributed manner to speed up the model training.

### 4.2. Datasets

(1) ExcelChart400K (EXC) [31]. This dataset was contributed by the work of ChartOCR. It contains a variety of charts including pie charts, bar charts, and line charts and was generated by the data of public Excel files. We selected 60,000 line chart images from the EXC dataset as the training set and 3000 images as the validation set.
(2) CHART2019-S (Chart2019). This dataset was generated using Matplotlib in the 2019 Harvest Primitive Tables competition, which contains six common chart types (e.g., bar, line, pie, etc.).
(3) FigureSeer (FS) [30]. This dataset was crawled from the CiteSeerX website, which contains more than 900 linear chart images. The annotation of FS contains the complete graphical information of the curve, giving the coordinates of each key on the curve, the position information of the text, and the position of the scale symbols, which was used by Clark and Divvala for curve tracing. Due to the limited data, we only used this dataset as a validation set.
(4) ICPR-2020-CHART-UB (PMC). This dataset contains 15 graph types, which were provided by the ICPR2020 CHART-Infographics competition, which are mainly from biomedical, material science, and other journals, and provide some manually annotated test samples. We filtered 1000 images with accurate annotations. Since these data are less annotated, we only use it as an evaluating dataset.

### 4.3. Evaluation Metrics

For each image, it is easy to be able to calculate the precision and recall of the predicted and true labeled images, which are calculated as follows:

(19) $$P=\frac{TP}{TP+FP},$$

(20) $$R=\frac{TP}{TP+FN},$$

where *TP* denotes the number of pixels predicted as curves with the true annotation as curves, *FP* denotes the number of pixels predicted as curves with the true annotation as background, and FN denotes the number of pixels predicted as background with the true annotation as curves. Then, we can easily calculate the F-measure ($2\cdot \frac{P*R}{P+R}$) of the model, which reflects the overall performance of the model.

Since our prediction result is a probability map and our task is similar to edge detection, we use the evaluation method of [23]. This evaluation method has two evaluation metrics: the global optimal F-measure, which is equal to the best F-measure with a fixed threshold on all images (ODS), and the local optimal F-measure, which is obtained from the best threshold for each image (OIS).

(21) $$ODS=\max\left\{2\frac{P_{t}*R_{t}}{P_{t}+R_{t}},\;t=\left(0.01,0.02,\cdots ,0.99\right)\right\},$$

(22) $$OIS=\frac{1}{N}\sum_{i}^{N}\max\left\{2\frac{P_{t}^{i}*R_{t}^{i}}{P_{t}^{i}+R_{t}^{i}},\;t=\left(0.01,0.02,\cdots ,0.99\right)\right\},$$

where *t* denotes the threshold; if the probability value is greater than *t*, it is represented as a curve, otherwise it is a background. $P_{t}$ and $R_{t}$ are the precision and recall of the threshold *t* on the entire dataset. $P_{t}^{i}$ and $R_{t}^{i}$ are the precision and recall for different thresholds *t* on image *i*. *N* is the number of images.

### 4.4. Comparison Methods

To validate the effectiveness of our proposed method, we compared it with classic edge detection algorithms HED and RCF, as well as semantic segmentation algorithms FCN and SegNet. Additionally, we included comparisons with the state-of-the-art algorithms for chart tasks, ChartOCR and LineEX. Previous methods for chart curve extraction primarily relied on color clustering techniques, which have significant drawbacks; therefore, we do not include these in our comparison. The descriptions of each algorithm are as follows:

(1) HED [17]. HED is an important breakthrough in edge detection, which is based on VGG16 implementation and can fuse multi-scale features. We trained HED on a mixed dataset, including EXC and Chart2019 datasets.
(2) RCF [18]. RCF is a modified version of HED that fuses multi-scale feature maps by convolution. Unlike HED, which chooses to take five side outputs, where each side output is the feature map of the convolution layer before the pooling layer, RCF takes the output feature map of each convolution as the side output, and RCF has a richer convolution feature. We trained RCF on mixed data, including EXC and Chart2019.
(3) FCN [16]. FCN is a multi-category semantic segmentation method. We modified the output layer and loss function of FCN to perform curve detection. The training and test sets are exactly the same as HED and RCF.
(4) SegNet [15]. SegNet builds encoding and decoding structures capable of end-to-end learning and segmentation, and we performed model modifications for curve detection. We trained on mixed data, including EXC and Chart2019.
(5) CS^2^Net [20]. CS^2^Net is a network for the linear structure segmentation of medical images that uses a pairwise attention mechanism to adaptively fuse global and local information. We retrained the model on the training set and tested the model effect.
(6) ChartOCR [31]. ChartOCR detects the curve key points and classifies the key points, and then implements the curve detection through the union–find algorithm. We used the model provided by [31] for testing.
(7) LineEX [32]. LineEX, similar to ChartOCR, performs curve detection through key point detection; however, it differs by using a Siamese neural network to group these key points. We used the model provided by [32] for testing.

### 4.5. Experimental Results

**Figure 5:**
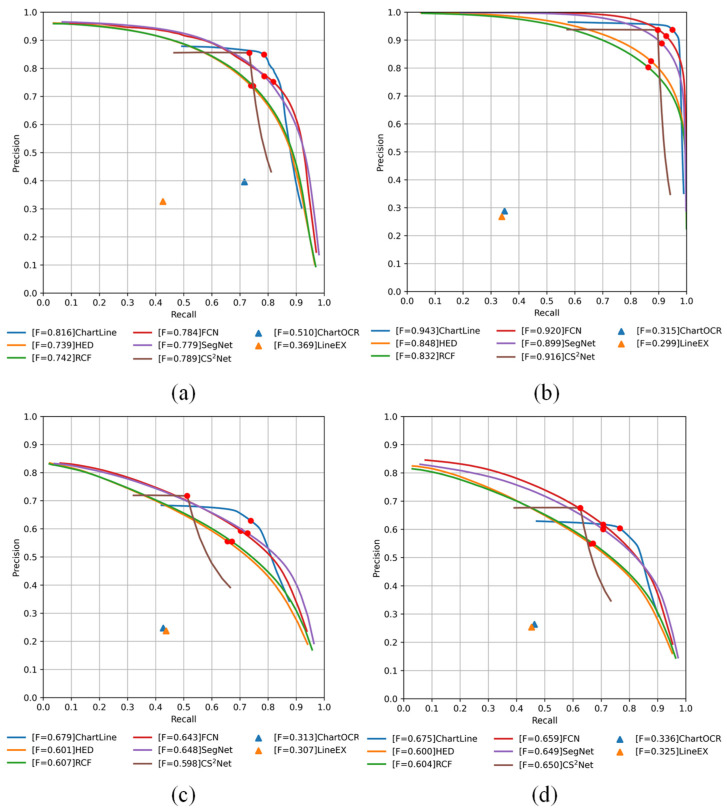
Precision–recall curves on the four test datasets. (**a**) EXC, (**b**) Chart2019, (**c**) FS, and (**d**) PMC. The red dots in the figure are the optimal values for each algorithm.

Figure 5 illustrates the precision–recall curves of seven methods on the four test datasets. Six methods are based on semantic segmentation to obtain a curve probability map with different precision and recall rates by selecting different thresholds t (0–1). A solid red circle is plotted at the position corresponding to the best F-measure for each curve. ChartOCR is based on a critical point and union–find algorithm to implement the reconstruction of curves, which produces complex curves; so, it is represented by a blue triangle on the image. LineEX and ChartOCR both utilize key points for detection, enabling curve detection by connecting these points. While their overall performances are similar, LineEX tends to have more false detections, which slightly reduces its overall curve detection accuracy, as shown by the orange triangles in Figure 5. In addition, we can see in Figure 5 that ChartOCR outperforms the other three datasets on the EXC dataset. ChartOCR is based on a model trained on EXC data, which is better adapted to that dataset and less adapted to types not seen before.

(1) EXC: As shown in Figure 5a, ChartLine has the best F-measure. The performance of HED and RCF are very similar, and the graphs show a large variation in accuracy and recall for both algorithms. A similar situation is observed for FCN and SegNet, where HED shows the lowest performance in deep learning for curve detection, suggesting that richer convolution features can improve curve detection performance. It can also be seen in Figure 5 that the decoder structure can significantly improve the performance of the model compared to models like HED and RCF, which have no decoding structure. In addition, by fusing the global and local information of the image, the curve detection performance can be effectively improved.

As shown in Table 1, ChartLine achieved the best performance with an F-measure of 0.8162. Compared to HED, RCF, FCN, SegNet, and CS^2^Net, our method improves ODS performance by 7.74%, 7.43%, 3.24%, 3.7%, and 0.27%, respectively. ChartOCR and LineEX exhibit similar performance, with the lowest OS across all datasets. However, their performance on the EXC dataset is significantly higher than that on the other three datasets. This is due to the fact that ChartOCR and LineEX are trained based on EXC data, which has relatively good adaptability on this dataset, and poor adaptability to types that have not been seen before.

(2) Chart2019: The Chart2019 image was generated using matplotlib, which has a cleaner background than the EXC dataset and a greater degree of curve differentiation; so, the semantic segmentation-based algorithm performs significantly better on the Chart2019 dataset than on the other three datasets, where ChartLine achieves the best performance. From Table 1, we observe that while FCN achieved the best results compared to HED, RCF, CS^2^Net, and SegNet, it is still 2.28% lower than our method in ODS and 2.39% lower in OIS. The ODSs of HED, RCF, CS^2^Net, and SegNet are 9.49%, 11.09%, 2.28%, and 4.4% lower than the results of ChartLine, respectively.
(3) FS: The images in this dataset are more difficult to extract than the previously mentioned datasets, and there is more noise near the curve, resulting in a lower detection accuracy than in the previous tests. As shown in Figure 5c and Table 1, ChartLine achieves the best performance, and the ODS reaches 67.94%. Compared to HED, RCF, FCN, SegNet, and CS^2^Net, the ODS of ChartLine is 7.82%, 7.21%, 3.62%, 3.13%, and 2.7% higher, respectively.
(4) PMC: The images in this test dataset are similar to the FS dataset in that they are all real images collected from papers, and the curves are more difficult to extract. Similar to the test results of FS, our method achieves the best ODS, which reaches 67.53%.

**Figure 6:**
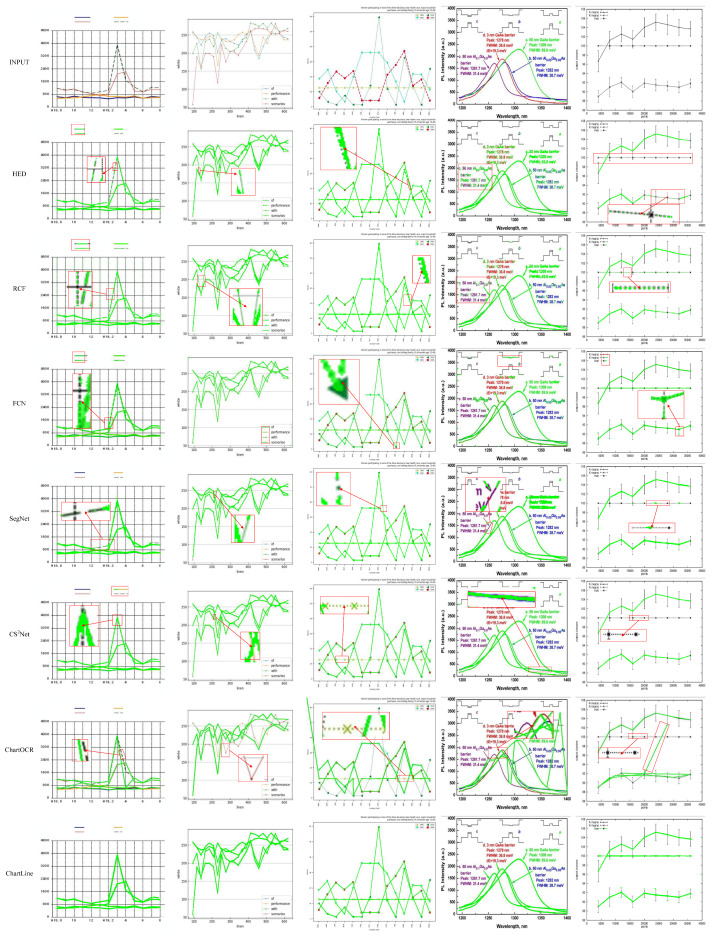
Curve detection results from seven different methods are shown across five types of line chart images. The top row displays the original source images, while each subsequent row presents the detection results from a different algorithm. Lines detected by each method are marked in green. Red boxes indicate specific errors, providing a zoomed view of areas where the detected lines and the true curves do not align.

For better comparison, we have selected different types of images from four datasets to visualize the results, as shown in Figure 6. The red rectangular box in Figure 6 shows the scenarios in which the comparison method predicts the wrong curve. From the images and results in the first column, we can see that ChartLine can filter the grid lines better, and the detection result is close to the natural label. Except for ChartOCR, other methods cannot eliminate the gridlines ideally, and the detection results produce more fine speckles. The second column shows an image with horizontal grid lines. Although each method achieves good results, the curves detected by all methods except ChartLine and ChartOCR have a lot of noise and discontinuities for the last two columns of curve images with different disturbances, which were real images from the FS and PMC datasets. ChartLine obtained results closer to the natural curve than the other methods. The comparison method, on the other hand, has more false predictions.

In summary, ChartLine achieves the best results on all test data by modeling global dependencies and locally linked sequences on the curve space and adaptive fusion of semantic features.

### 4.6. Ablation Study

We conducted ablation studies on all modules of ChartLine, using a structure that includes a feature pyramid as the baseline. We then evaluated the impact of the Spatial-Sequence Fusion (SSF) module and the Channel Multi-Head Attention (CMA) module on the model performance. For clarity, we referred to these as ChartLine-SSF and ChartLine-CMA, respectively. Additionally, we compared the hybrid loss (HL) with the binary cross-entropy (BCE) loss function. For details on SSF, CMA, and HL, please refer to Section 3.2, Section 3.3 and Section 3.4. The results of the ablation experiments are shown in Table 2.

**Figure 7:**
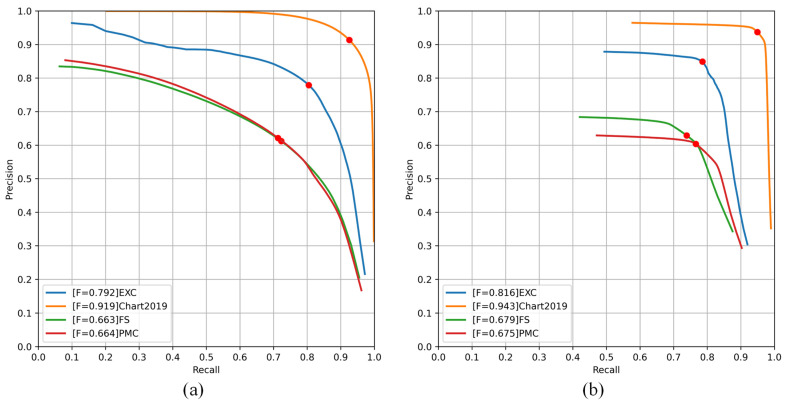
P-R curves with different loss functions on four datasets. (**a**) The result of ChartLine-BCE. (**b**) The result of ChartLine.

- **Spatial-Sequence Fusion (SSF) module:** In Table 2, we can see that our proposed SSF module can significantly improve the performance of the curve detection. Compared to the baseline, SSF improves the ODS on the EXC, Chart2019, FS, and PMC datasets by 1.8%, 3.9%, 1.21%, and 0.58%, respectively.
- **Channel Multi-Head Attention (CMA) module:** From Table 2, we can see that the Channel Multi-Head Attention (CMA) module also enhances the baseline performance. On the synthetic datasets EXC and Chart2019, ODS improved by 1.59% and 3.71%, respectively, while on the real datasets FS and PMC, ODS increased by 0.95% and 0.22%, respectively.
- **Hybrid loss (HL) function:** Compared to ChartLine-BCE, we can see that our proposed hybrid loss function can effectively improve the performance of the model, compared to the common cross-entropy loss function, with a 1–2% performance improvement on the four test datasets. In addition, we have also visualized the different P-R curves, and Figure 7 fully demonstrates that our proposed loss function has a stable precision and recall due to the ability of the model to adjust for incorrectly predicted samples during the training process.

**Figure 8:**
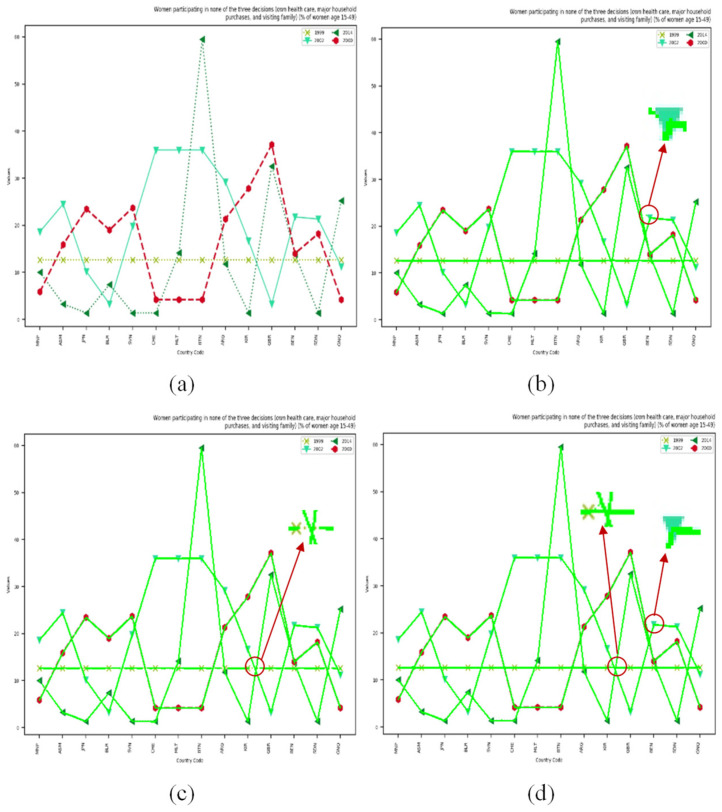
The detection results of different modules: (**a**) the raw image, (**b**) the detection result of ChartLine-SSF, (**c**) the detection result of ChartLine-CMA, and (**d**) the detection result of ChartLine. The red circles in the image zoom in on local details for easy comparison.

In addition, by comparing the data of ChartLine-SSF, ChartLine-CMA, and ChartLine in the table, we can further conclude that the combination of SSF and CMA enhances baseline performance. The SSF module improves the continuity of the curves, while the CMA module differentiates between the background and the curves adaptively through the self-attention mechanism. The combination of these two effectively enhances the model’s performance. To better demonstrate the performance of the different modules on the model, the experimental results are visualized in Figure 8. In Figure 8b, the red markers indicate that using only the SSF module leads to background prediction errors, which do not accurately identify the boundaries of the curves. In Figure 8c, the red markers show that using only the CMA module results in local discontinuities. Figure 8d demonstrates that combining SSF and CMA effectively improves model accuracy.

### 4.7. Running Efficiency

We test the inference time of the compared methods on all datasets. As shown in the last column of Table 1, ChartLine and the other four methods can detect curves in line chart images efficiently. ChartLine processes a 512 × 512 line chart image at 20 FPS. SegNet is faster than ChartLine, and takes 50 ms per image. HED, RCF, and FCN, with fewer parameters, can achieve faster speeds of about 42 FPS, 35 FPS, and 40 FPS. ChartOCR and LineEX are two-stage curve detection algorithms that rely on critical point grouping to achieve curve detection; so, we did not compare ChartOCR and LineEX with our proposed ChartLine.

## 5. Conclusions

In this work, we propose a novel model for curve detection, ChartLine, which consists of an encoder–decoder structure and an SSA-FPN, where SSA consists of a Spatial-Sequence Fusion (SSF) module and a Channel Multi-Head Attention (CMA) module. In ChartLine, top-down multi-scale convolutional features are fused to better accommodate the delicate features of curves. The SSA-FPN enhances the curve representation capability, where SSF achieves long-range dependence modeling and local representation capability to enhance the continuity of curves. CMA adaptively fuses local and global semantic information to reduce the recognition error of curves. In addition, a hybrid loss function is proposed in this paper, which effectively solves the problem of imbalance between positive and negative samples of curves and improves the accuracy and stability of the model. We collected and constructed four line-chart datasets for experiments and evaluations. The experimental results show that the proposed ChartLine achieves more than 94% F-values on the noise-interference-free color line graph images (Chart2019), which exceeds the current state-of-the-art methods.

## Figures and Tables

**Table 1:** Quantitative evaluation of different methods on four test datasets. ChartOCR and LineEX, as key point detection algorithms, achieve curve extraction through key grouping without threshold settings; therefore, they only have the OIS metric, while the ODS is represented by “-”.

Methods	EXC		Chart2019		FS		PMC		FPS
	ODS	OIS	ODS	OIS	ODS	OIS	ODS	OIS	
HED	0.7388	0.7520	0.8482	0.8619	0.6012	0.6213	0.6003	0.6306	42
RCF	0.7419	0.7534	0.8322	0.8435	0.6073	0.6287	0.6042	0.6322	35
FCN	0.7838	0.8107	0.9203	0.9292	0.6432	0.6514	0.6592	0.6596	40
SegNet	0.7792	0.7960	0.8991	0.9108	0.6481	0.6621	0.6492	0.6574	25
CS^2^Net	0.7892	0.8024	0.9101	0.9212	0.5977	0.6215	0.6503	0.6621	24
ChartOCR	-	0.5097	-	0.3154	-	0.3131	-	0.3031	-
LineEX	-	0.3691	-	0.2992	-	0.3075	-	0.3000	-
ChartLine	0.8162	0.8233	0.9431	0.9531	0.6794	0.6892	0.6753	0.6820	20

**Table 2:** Quantitative evaluation using different modules of ChartLine on four datasets. “×” indicates that the module is not used and “√” signifies that the module is used.

Methods	Modules			EXC		Chart2019		FS		PMC	
	SSF	CMA	HL	ODS	OIS	ODS	OIS	ODS	OIS	ODS	OIS
Baseline	×	×	√	0.7851	0.8014	0.9030	0.9181	0.6575	0.6682	0.6686	0.6712
ChartLine-SSF	√	×	√	0.8031	0.8175	0.9420	0.9528	0.6696	0.6722	0.6744	0.6813
ChartLine-CMA	×	√	√	0.8010	0.8141	0.9401	0.9511	0.6670	0.6812	0.6708	0.6800
ChartLine-BCE	√	√	×	0.7917	0.8015	0.9194	0.9256	0.6630	0.6711	0.6642	0.6794
ChartLine	√	√	√	0.8162	0.8333	0.9431	0.9531	0.6794	0.6992	0.6753	0.6820

## Data Availability

Data is contained within the article.
